# COVID-19 Contact Tracing in a Pediatric Hospital: Maximizing Effectiveness Through Specialized Team and Automated Tools

**DOI:** 10.1017/ash.2021.86

**Published:** 2021-07-29

**Authors:** Lindsay Weir, Jennifer Ormsby, Carin Bennett-Rizzo, Jonathan Bickel, Colleen Dansereau, Matthew Horman

## Abstract

**Background:** In their interim infection prevention and control recommendations for the coronavirus disease 2019 (COVID-19) pandemic, the Centers for Disease Control and Prevention (CDC) recommend that healthcare facilities have a plan to identify, investigate, and trace potential COVID-19 exposures. In an academic hospital, the scale of such tracing is substantial, given that medically complex patients can have dozens of staff contacts across multiple locations during an encounter. Furthermore, the family-centered care model employed by pediatric institutions precludes visitor exclusion, further complicating tracing efforts. Despite this complexity, tracing accuracy and timeliness is of paramount importance for exposure management. To address these challenges, our institution developed a contact-tracing system that balanced expert participation with automated tracing tools. **Methods:** Our institution’s contact-tracing initiative includes positive patients, parents and/or visitors, and staff for the enterprise’s inpatient, procedural, and ambulatory locations at the main campus and 4 satellites. The team consists of 11 staff and is overseen by an infection preventionist. For positive patients and parents and/or visitors, potentially exposed staff are automatically identified via a report that extracts staff details for all encounters occurring during the patient’s infectious period. For positive staff, trained contact tracers call the staff member to determine whether mask and distancing practices could result in others meeting CDC exposure criteria. Any potentially exposed healthcare workers (HCWs) receive an e-mail that details exposure criteria and provides follow-up instructions. These HCWs are also entered into a secure, centralized tracking database that (1) allows infection prevention and occupational health staff to query and identify all epidemiologic links between traced patients, parents and/or visitors, and staff, and (2) initiates staff enrollment in a twice-daily symptom tracking system administered via REDCap. Potentially exposed patients and parents and/or visitors are contacted directly by a hospital representative. The contact tracing team, infection prevention staff, and occupational health staff meet daily to review positive staff cases in the last 24 hours. **Results:** To date, the team has traced ~1,300 patients, 15 parents and/or visitors, and 700 staff. Since the start of the pandemic, tracing and contact notification for all positive cases has been conducted within 24 hours. Through these proactive tracing efforts and other institutional infection prevention initiatives, the institution only experienced 1 staff cluster (N < 15) and <5 hospital-onset patient cases. **Conclusions:** Equipping a trained group of contact tracers with automated tracking tools can afford infection prevention and occupational health departments the ability to achieve and sustain timely and accurate contact tracing initiatives throughout a large-scale pandemic response.

**Funding:** No

**Disclosures:** None

